# Supplementary material for: Human Synovial Mesenchymal Stem Cells Expressed Immunoregulatory Factors IDO and TSG6 in a Context of Arthritis Mediated by Alphaviruses
Source: Int J Mol Sci. 2023 Nov 3;24(21):15932. doi: 10.3390/ijms242115932 (PMC10649115; doi:10.3390/ijms242115932)
Supplement: Supplementary file 1 [file ijms-24-15932-s001.zip › ijms-2664607-supplementary.pdf]

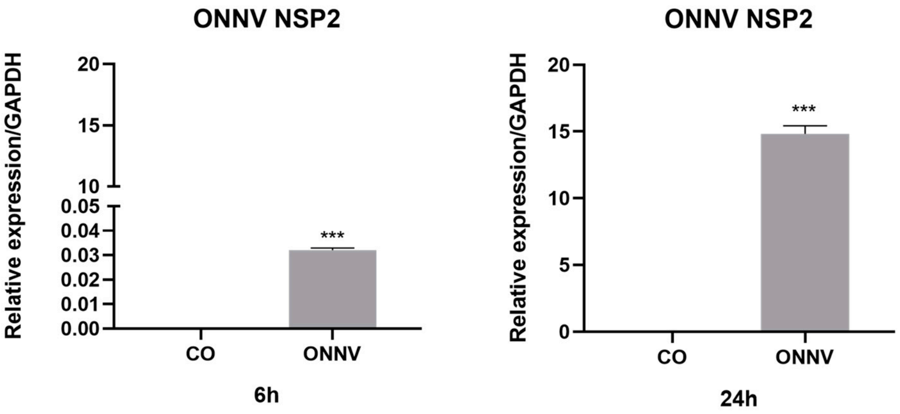

Figure S1: NSP2 ONNV was increased in synovial tissue-derived MSCs infected with ONNV.

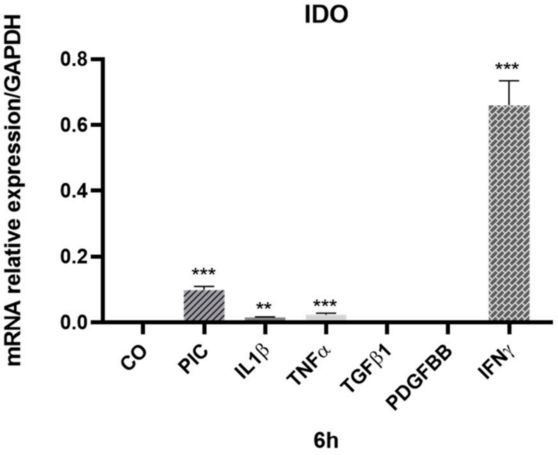

Figure S2: Regulation of *IDO* and *TSG6* expression in an in vitro model of arthritis.

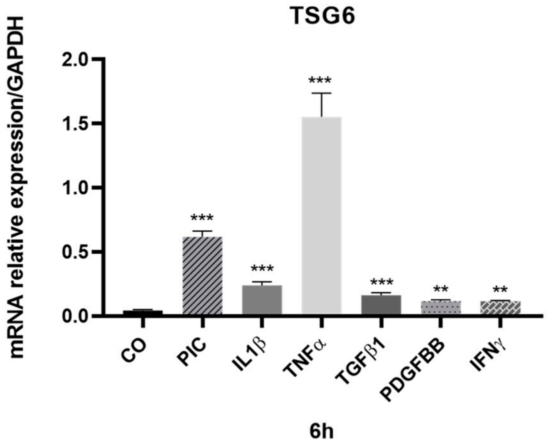

Figure S3: Synovial tissue-derived MSCs expressed *MXRA8*.

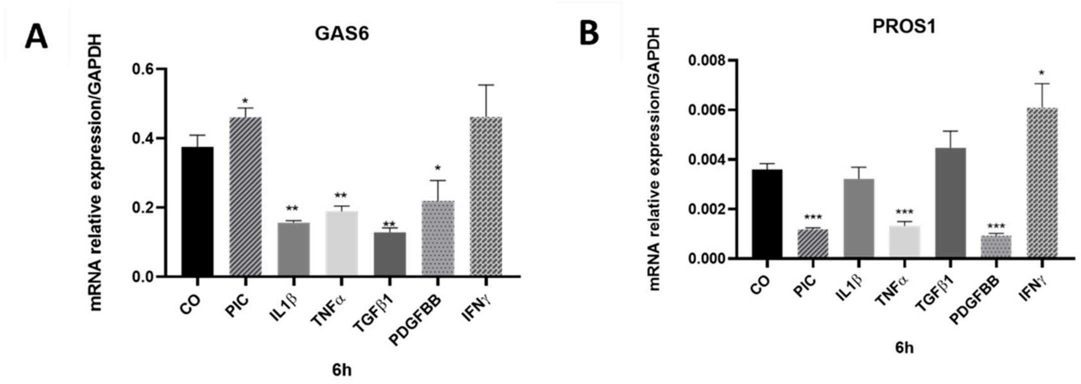

Figure S4: Regulation of *GAS6* and *PROS1* expression in an *in vitro* model of arthritis.
